# Taurine and Its Derivatives: Analysis of the Inhibitory Effect on Platelet Function and Their Antithrombotic Potential

**DOI:** 10.3390/jcm11030666

**Published:** 2022-01-27

**Authors:** Adrian Eugen Roşca, Ana-Maria Vlădăreanu, Radu Mirica, Cristina-Mihaela Anghel-Timaru, Alina Mititelu, Bogdan Ovidiu Popescu, Constantin Căruntu, Suzana Elena Voiculescu, Şerban Gologan, Minodora Onisâi, Iuliana Iordan, Leon Zăgrean

**Affiliations:** 1 Department of Physiology, “Carol Davila” University of Medicine and Pharmacy, 050474 Bucharest, Romania; cristina.timaru@umfcd.ro (C.-M.A.-T.); costin.caruntu@gmail.com (C.C.); suzana.voiculescu@umfcd.ro (S.E.V.); leon.zagrean@umfcd.ro (L.Z.); 2 Department of Cardiology, Emergency University Hospital of Bucharest, 050098 Bucharest, Romania; 3 Department of Hematology, “Carol Davila” University of Medicine and Pharmacy, Emergency University Hospital of Bucharest, 050098 Bucharest, Romania; alina.mititelu@drd.umfcd.ro (A.M.); minodorel@yahoo.com (M.O.); iuliana.iordan@drd.umfcd.ro (I.I.); 4 Department of Surgery, “Carol Davila” University of Medicine and Pharmacy, “Sf. Ioan” Clinical Hospital, 042122 Bucharest, Romania; mirica_rm@yahoo.com; 5 Department of Neurology, “Carol Davila” University of Medicine and Pharmacy, Colentina Clinical Hospital, 020125 Bucharest, Romania; bogdan_ovidiu_popescu@yahoo.com; 6 Department of Dermatology, “Prof. N.C. Paulescu” National Institute of Diabetes, Nutrition and Metabolic Diseases, 011233 Bucharest, Romania; 7 Department of Gastroenterology, “Carol Davila” University of Medicine and Pharmacy, Elias Clinical Hospital, 011461 Bucharest, Romania; serbangologan@gmail.com; 8 Department of Medical Semiology and Nephrology, “Carol Davila” University of Medicine and Pharmacy, 050474 Bucharest, Romania

**Keywords:** taurine, taurine derivatives/analogues, hemostasis/haemostasis, platelet activity, platelet reactivity, platelet aggregation, coagulation, thrombosis, prothrombotic state, thrombotic diathesis

## Abstract

Taurine is a semi-essential, the most abundant free amino acid in the human body, with a six times higher concentration in platelets than any other amino acid. It is highly beneficial for the organism, has many therapeutic actions, and is currently approved for heart failure treatment in Japan. Taurine has been repeatedly reported to elicit an inhibitory action on platelet activation and aggregation, sustained by in vivo, ex vivo, and in vitro animal and human studies. Taurine showed effectiveness in several pathologies involving thrombotic diathesis, such as diabetes, traumatic brain injury, acute ischemic stroke, and others. As human prospective studies on thrombosis outcome are very difficult to carry out, there is an obvious need to validate existing findings, and bring new compelling data about the mechanisms underlying taurine and derivatives antiplatelet action and their antithrombotic potential. Chloramine derivatives of taurine proved a higher stability and pronounced selectivity for platelet receptors, raising the assumption that they could represent future potential antithrombotic agents. Considering that taurine and its analogues display permissible side effects, along with the need of finding new, alternative antithrombotic drugs with minimal side effects and long-term action, the potential clinical relevance of this fascinating nutrient and its derivatives requires further consideration.

## 1. Introduction

Taurine (2-aminoethanesulfonic acid) is a phylogenetically ancient compound with a large distribution in the biosphere, present in high concentration in algae and animals, including insects and arthropods, but generally absent or nearly absent in bacteria and plants. In many animals, including mammals, it is the most abundant low-molecular-weight organic constituent. A 70 kg human body contains up to 70 g of taurine [1,2]. Although an amino acid, it is not used in protein synthesis [3].

High concentrations in humans are found in bile, myocardium, skeletal muscles, liver, nervous system, intestine, kidney, retina, and blood cells (leukocytes, platelets) [4,5]. The endogenous synthesis from methionine and cysteine is insufficient, therefore diet remains the major source of taurine, especially seafood, eggs, and meat. Taurine has a proven benefit as a pharmaconutrient or a conditionally essential amino acid, being administered to subjects requiring long term parenteral nutrition, including newborns or premature infants. It has also gained more and more popularity as an ingredient in food supplements and energy drinks [6,7,8].

An important step in understanding the action of taurine was reached in 1968, along with the publication of Jacobsen and Smith’s comprehensive review [1]. Until that point all known data about its action were reduced to bile salt synthesis, osmoregulation in marine invertebrates, energy storage in marine worms, and neural inhibition in the central nervous system. Since then, the range of its physiological functions has considerably expanded. Nowadays, there is overwhelming evidence about the involvement of taurine in fundamental physiological processes, such as neuromodulation, bile acid conjugation, intestinal microbiota homeostasis, regulation of energy metabolism, muscle contraction, ion transport, calcium handling, immunomodulation, anti-inflammatory and anti-oxidative response, osmoregulation, and cell membrane stabilization and apoptosis [3,4,9,10,11]. Taurine has been further proved to exert many pharmacologic actions, acting as a protective agent against pathologies including nervous diseases (retinal degeneration, stroke, neurodegenerative diseases—Parkinson’s, Alzheimer’s, or Huntington’s disease—epilepsy, fragile X syndrome, succinic semialdehyde dehydrogenase deficiency), metabolic diseases (diabetes mellitus, stroke-like episodes - MELAS, mitochondrial disease), inflammatory diseases, sarcopenia, myotonic dystrophy, and Duchenne muscular dystrophy [9,10,11]. Moreover, taurine elicits protection against cardiovascular disease [5,9,12,13,14], delaying the progression of atherosclerosis [15,16], reducing blood pressure [17,18,19,20], preventing the development and correcting cardiomyopathy [21,22,23,24,25,26], showing efficacy in ischemia-reperfusion injury [9,27], manifesting antiarrhythmic properties, preventing sudden death [28,29,30,31,32], and acting as a cardioprotective agent in congestive heart failure (CHF) [11,33]. As a matter of fact, taurine has been approved to be included in the treatment of CHF in Japan [9]. Generally, no side effects of taurine administration have been reported at regular doses, nor major ones at high doses (over 6 g/day) [8,11,33,34,35,36]. Through all these above mentioned actions, taurine can represent an important future component of the newer promising therapies against cardiovascular disease.

Last but not least, taurine has been proven to manifest inhibitory effects on key processes of hemostasis, such as platelet activation and aggregation. Our descriptive article aims to review available data regarding the influence of taurine and its derivatives on platelet function, focusing on its claimed antithrombotic potential.

## 2. Overview of the Antithrombotic Potential of Taurine

Initial data suggesting a likely antithrombotic role of taurine came from observational studies which identified high levels of taurine in hibernating animals while acclimating to hypothermic conditions. Therefore, taurine has drawn attention through its possible role in freeze tolerance. The hibernating animals are at risk for intravascular thrombosis due to very slow blood flow and increased blood viscosity induced by low temperatures exposure. It has been shown that the total amino acid pool increased more than two-fold in hibernating animals (hatchling turtles, frogs) with taurine accounting for about 50% of this increase [37,38]. Moreover, a decrease in total amino acid plasma levels has been shown in the process of snakes’ cold acclimation, except for taurine (the single one that increased). Clotting times prolongation has been recorded when blood humoral coagulation of these animals was assessed [38,39,40]. All these led to an interesting emerging hypothesis regarding the possible role of taurine in preventing blood clotting [37,38].

Next, in vivo experiments have been developed in order to explore the putative anti-thrombotic activity of taurine. Huang and Rao studied the effect of taurine, separately and in combination with neferine, on platelet aggregation and thrombus formation in rats. Both drugs have been found to inhibit platelet aggregation triggered by various agonists when assessed by turbidimetry, and taurine (100 mg/kg) reduced the wet weight of induced thrombosis by a rate of 47.82% vs. control rats [41].

In another rat model of arterial thrombosis (ferric trichloride injection in the abdominal aorta), taurine enhanced urokinase (UK)-induced arterial recanalization compared to UK alone, as concomitant administration of drugs reduced the area of the thrombus cross-section [42]. Later, Murina et al. performed an in vivo experiment of arterial thrombosis [43] using a taurine derivative, N,N-dichlorotaurine (DT), a compound synthesized via the reaction of taurine with hypochlorite (which is produced in vivo by activated neutrophils in myeloperoxidase-catalyzed reactions). Taurine can act as a trap for “active chlorine”, attenuating the oxidative damage induced by hypochlorite and providing cell protection. But most of all, the taurine analog DT seemed to manifest remarkable antithrombotic properties. Intravenous injection of DT (3.4–6.8 mg/kg, with a blood DT concentration of 0.25–0.5 mM) 10 min prior to the intravenous injection of the thrombotic agent ADP (300 mg/kg), resulted in a reduced mortality rate (up to 10%, compared to 96% in controls), and a locomotor activity of the survivors returning to normal in 1 to 5 min after the treatment. In terms of mortality rate, this result appeared to be more valuable than that obtained using ASA (20 mg/kg, blood concentration of 1.7 mM) or ticlopidine (10 mg/kg, blood concentration of 0.5 mM), but unfortunately, it lacked statistical power. In a second series of experiments, DT (6.8 mg/kg) administered before injecting an epinephrine-collagen mixture (8.6 mg/kg epinephrine, concomitantly with 15 mg/kg collagen) was shown to be effective in increasing survival rate from 16% in controls to 64% in treated rats. Considering that ASA and ticlopidine doses used in this study were similar to those recommended for a single week of oral treatment, and that used DT doses were lower than those causing acute toxicity (the lethal doses for 50% of mice were 48 mg/kg, by one order higher magnitude than the therapeutic one), the authors speculated that DT may represent a promising antithrombotic agent [43].

An in vivo rat model of thrombosis using a combination of stasis and hypercoagulability in the inferior vena cava showed lower efficacy in preventing thrombus formation of another taurine derivative, taurolidine (10 mg, or 20 mg, i.v) when compared to low-molecular weight heparin (nadroparin calcium, 100 antiXa ICU/2 mL/kg). Thrombus weight was significantly lower in the taurolidine-treated group than in controls (a decrease of 42%) but remained significantly higher than in the heparin-treated group. It has also been noted that taurolidine elicits only a limited influence on the activity of several plasma coagulation factors (significant reduction of factors V, VIII, IX, XI and XII activities, and insignificant reduction of factors II, VII and X activities), without changing prothrombin time, or activated partial thromboplastin time. The conclusion was that taurolidine is more likely to be ineffective in venous thrombosis prevention in this experimental setting [44].

Based on the concept that atheroembolic lesions dissolution might require both an antithrombotic agent and a lipid emulsifier, a new idea emerged in the 1990s concerning the neuroprotective effects of taurine and its derivatives on ischemic brain damage. Jeynes et al. demonstrated in two successive experiments that combined treatment with taurochenodeoxycholate and streptokinase can reduce the size of in vitro generated thrombi, and dramatically reduce the area and perimeter of the in vivo infarcts in rabbits subjected to cerebral-induced atherothromboembolism [45,46]. Moreover, Sun et al. showed that taurine can decrease cerebral infarct volume in rats who underwent transient middle cerebral artery occlusion (MCAo) in a dose-dependent manner (5, 15, 50 mg/kg), pointing caspase-3 and calpain-mediated apoptosis blockage as one of the possible mechanisms for its protective role against focal cerebral ischemia [47]. The authors further reported reduced ischemic brain damage with taurine administration (50 mg/kg, i.v) in a rat model of a stroke, and newly pointed taurine might act via suppression of Poly (ADP-ribose) polymerase (PARP) and nuclear factor-kappaB (NF-κB) pathways [48]. The same research group also demonstrated that taurine and urokinase co-administration after experimental MCAo in rats can widen the therapeutic window, without augmenting the hemorrhagic transformation [49]. They suggested the extension of the therapeutic window following delayed intravenous administration of taurine (50 mg/kg) may continue up to 8 h after the experimental induction of stroke [50]. Later, Rukan et al. noted that taurine administration after ischemic-reperfusive brain damage can restore normal endothelial function, inhibit platelet activity and humoral coagulation, and remove brain morphological changes in rats [51]. Gharibani and colleagues concomitantly reported the efficacy of taurine administration in a model of focal rat MCAo, showing a great reduction of the infarcted area, and neuroprotective effects via inhibition of apoptosis and downregulation of the activating transcription factor 6 (ATF6) and inositol requiring enzyme 1 (IRE-1) pathways [52]. Similar effects of taurine on infarct size have been noted following the combined treatment with taurine (40 mg/kg) and S-Methyl-N,N-diethylthiolcarbamate sulfoxide (DETC-MeSO) in transient MCAo, which led to a synergistic suppression of all three pathways of endoplasmic reticulum (ER) stress-induced apoptosis, suggesting an effective novel treatment that might be used in ischemic stroke [53].

Recently, Jin et al. investigated the effect of taurine treatment after thrombolytic therapy with tissue-type plasminogen activator (tPA) in a rat MCAo model. Taurine (50 mg/kg i.v, at 4 h, for 3 days) reduced the infarct size versus saline at 4 h after the induced stroke, while the combined treatment with taurine (given at the same time-dose) and delayed tPA (10 mg/kg, at 6 h) was more effective than tPA therapy alone (10 mg/kg, at 2 h). Taurine has also been proved to markedly reduce the intravascular fibrin depositions and thrombocytes accumulation in downstream microvessels (inhibiting secondary thrombus formation associated with early reperfusion). Therefore, taurine may enhance the efficacy of thrombolysis, and may extend the therapeutic window duration of tPA from 2–3 h to 6 h after embolic MCAo in rats. Additionally, taurine administered in combination with delayed t-PA has been shown to profoundly prevent tPA-associated hemorrhage (6 h after the onset of ischemia), to reduce mortality rate at 3 days from 47% to 21%, to significantly improve the long-term outcome after stroke, and to reduce the 45-day mortality rate from 55% to 31% (following 7 days taurine administration). Taurine seems to reduce tPA-associated hemorrhage acting through a profound inhibition of CD147 (cluster of differentiation 147)-dependent MMP-9 (matrix metalloproteinase-9) pathway in the ischemic brain endothelium [54]. On the other hand, it is well known that CD147 is involved in intravascular fibrin and platelet deposition, by interacting with platelet GP VI (glycoprotein VI), and its inhibition has been proven to reduce microvascular thrombosis in acute ischemic stroke [55,56,57]. Thereby, as suggested by Jin et al. [54] and previously by Sun M. et al. [48], intense downregulation of CD147 expression, and inhibition of NF-κB activation by taurine may underlie its beneficial influence in terms of improving the cerebral microvascular patency following tPA thrombolysis in rat embolic MCAo. Jin and colleagues concluded that taurine exerts potent anti-thrombotic effects, and the combination of taurine and tPA may be clinically relevant as a new future strategy in embolic stroke therapy.

Animal models of thrombosis seem to provide consistent evidence regarding the antithrombotic potential of taurine and its derivatives. However, further preclinical experiments need to be carried out, in order to produce a natural transition towards clinical trials. Due to ethical considerations, although highly needed, prospective human studies on thrombosis outcomes are very difficult to achieve. Up-to-date knowledge regarding the overall assessment of taurine effectiveness in human thrombotic conditions is only based on few observational and prospective-case control trials. One of these studies is the one published by Ijiri et al. in 2013, investigating the antithrombotic effect of the sulfur-amino acid on 101 enrolled healthy Japanese volunteers reporting a taurine-rich diet. The protocol used a new point-of-care test (a Global Thrombosis Test-GTT) that allowed the assessment of the entire hemostatic process (platelet reactivity, humoral coagulation, and endogenous thrombolytic activity). While nonsignificant correlations have been observed between taurine concentration in urine samples and GTT-Occlusion Times measured in nonanticoagulated blood samples of volunteers, a significant inverse correlation has been registered between urinary taurine concentration and GTT-Lysis Times, suggesting that a taurine-rich diet may enhance spontaneous thrombolytic activity [58]. Since 1985, the same research group has conducted several multicenter epidemiological studies based on data from World Health Organization (WHO) surveys on diets and cardiovascular disease (CVD) risk and mortality (the largest covering 61 populations in 25 countries, including men and women). Urinary taurine excretion per 24 h (24-UT) has been used in these trials as a biomarker for dietary taurine intake. A significant inverse correlation between 24-UT and mortality rate from stroke and coronary heart disease (CHD) has been reported [59,60,61,62,63,64]. On the other hand, a prospective nested case-control study, including 14,274 women from a breast cancer screening center enrolled since 1985 and until 1991, revealed no statistically significant association between serum taurine and stroke risk. The aforementioned epidemiological study noted however a significant inverse correlation of these parameters in the never-smokers subgroup, information deserving further consideration [65].

Direct human data related to the possible anti-thrombotic role of taurine in patients with thrombosis are scarce, due to methodological limitations. However, there is a considerable larger amount of indirect data coming from human and animal studies assessing taurine and its derivatives’ action on a distinct key process of hemostasis - platelet activation, with subsequent aggregation. These studies will be discussed in the next sections.

Besides taurine influence on hemostasis *per se*, its beneficial action in terms of shifting hemostatic balance to a lower hypercoagulant status may be also linked to its favorable influence on cardiovascular risk markers. Taurine has been proved to delay atherosclerosis onset by diminishing cholesterol biosynthesis rate, decreasing hepatic and serum cholesterol level, reducing hepatic biosynthesis of cholesterol esters and triglycerides, decreasing hyperhomocysteinemia, suppressing lipoxygenase 1 (LOX-1) expression, protecting endothelial cells from the toxicity generated through oxidation and glycation of LDL, downregulating vascular smooth muscle cell (VSMC) proliferation through platelet-derived growth factor-BB (PDGF-BB) inhibition, increasing vascular production of nitric oxide, and generally attenuating the neointimal inflammation and oxidative stress [11,16,66,67,68,69,70,71,72,73,74,75,76,77,78,79]. Apart from the protective effect regarding dyslipidemia, taurine also positively influences other components of metabolic syndrome (obesity, hypertension, and diabetes), and even showed effectiveness in combating diabetes complications, including nephropathy, retinopathy, neuropathy, and cardiomyopathy [17,20,79,80,81,82,83,84,85]. Because these cardiovascular risk markers have also been independently associated with coagulation abnormalities, such as enhanced platelet aggregation, activated humoral coagulation, or suppressed fibrinolysis [86,87,88,89,90,91,92,93,94,95,96], there is reason to believe the antithrombotic properties of taurine could partially be ascribed to its beneficial influence on these conventional cardiovascular risk factors. Finally, taurine has the ability to induce structural “reverse-remodeling” following myocardial injury, reducing oxidative stress, apoptosis, myocardial hypertrophy and fibrosis as a reparative process [27,97,98,99,100,101,102]. Thereby, taurine contributes to the attenuation of the functional (electrical) remodeling and diastolic function impairment, leading to a reduced risk for arrhythmias onset [29,30,31,32,103], which would otherwise predispose to intracavitary thrombosis and further systemic embolism.

## 3. Overview of Platelet Function

**Figure 1:**
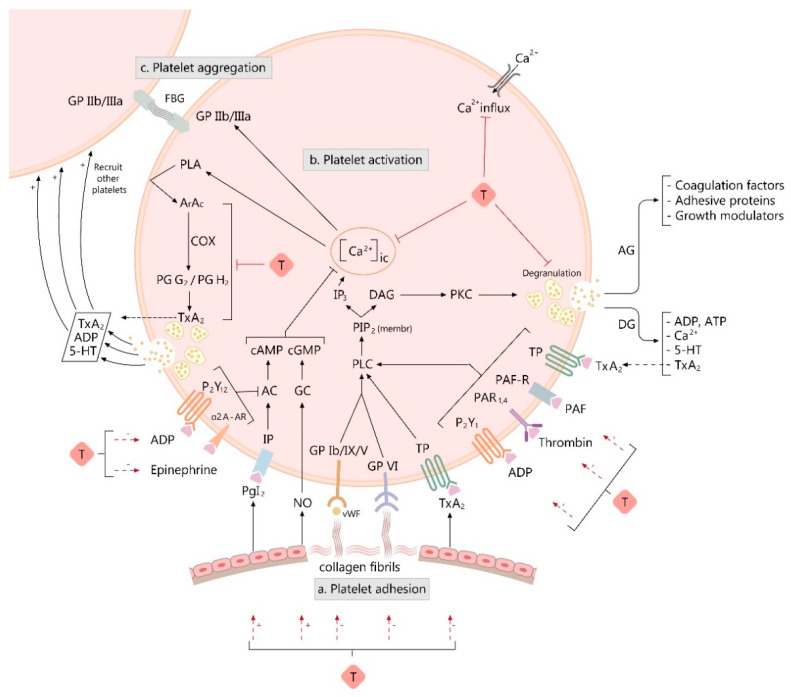
Figure illustrating the influence of taurine and its derivatives (T) on various processes involved in platelet activation and aggregation. The proposed mechanisms of action (indicated by red arrows) are the following: 1. T inhibits platelet aggregation triggered by collagen, which is a strong platelet activator, but also an adhesion surface, being responsible for *platelet adhesion* (**a**); 2. T inhibits *platelet activation and secretion* (**b**) and consequently the process of *platelet aggregation* (**c**) by: (1) controlling intracellular calcium concentration-[Ca^2+^]_ic_ (limiting the calcium influx and suppressing the intraplatelet calcium (Ca^2+^) response to activating agonists); (2) down-regulating a growth modulator, β-thromboglobulin (β-TG) and adenosine triphosphate (ATP) release response to agonists, as markers of discharge from alpha and dense platelet granules; (3) decreasing the platelet cyclooxygenase activity and the platelet thromboxane A_2_ (TxA_2_) production; (4) suppressing platelet aggregation triggered by various agonists, such as adenosine diphosphate (ADP), thrombin, epinephrine, or platelet activation factor (PAF); (5) increasing of the endothelial nitric oxide (NO) release and prostacyclin (PgI_2_) production and decreasing TxA_2_ release. Other used abbreviations: GP—glycoprotein; vWF—von Willebrand factor; FBG—fibrinogen; PLC—phospholipase C; PIP_2_ (membr)—phosphatidylinositol 4,5-bisphosphate from platelet plasmalemma; IP_3_—inositol 1,4,5-trisphosphate; DAG—1,2-diacylglycerol; PKC—protein kinase C; DG—dense granule; AG—alfa-granule; 5-HT—5-hydroxytryptamine; PAF-R—PAF receptor; PAR_1,4_—protease-activated receptor-1 and -4; P2Y_1_—purinergic receptor 2Y_1_; P2Y_12_—purinergic receptor 2Y_12_; IP—prostacyclin receptor; TP—TxA_2_ receptor; α2 A-AR—alpha 2A adrenergic receptor; AC—adenylyl cyclase; cAMP—cyclic adenosine monophosphate; GC—guanylate cyclase; cGMP—cyclic guanosine monophosphate; PLA—phospholipase A; ArAc—arachidonic acid; COX—cyclooxygenase; PgG_2_—prostaglandin G_2_; PgH_2_—prostaglandin H_2_; (+) on the arrow represents up-regulation, (−) on the arrow represents down-regulation.

The involvement of platelets in normal hemostasis and vascular disease is well known. Platelets’ functions in hemostasis and thrombosis have been extensively studied in the past decade, providing a valuable insight into the elements and signals regulating platelet adhesion, activation and aggregation [104]. For an efficient hemostasis, it is mandatory that the mechanisms leading to platelet adhesivity, granule release, platelet activation and aggregation happen simultaneously (Figure 1).

Following blood vessel injury, platelets interact with collagen fibrils in the exposed subendothelium by a process *(adhesion)* that involves, among other events, the interaction of von Willebrand factor (vWF) plasma protein with a specific glycoprotein (GP) complex on the platelet surface, GP Ib/IX/V. This promotes direct interaction of collagen receptors (GPVI and α2β1 integrin) on platelets with collagen fibrils from the subendothelial matrix, initiating the process of platelet activation. Collagen is an important participant, as it represents both an adhesion surface and a strong platelet activator [105].

*Platelet activation and granule release* are influenced by humoral mediators (platelet activating factor—PAF, thrombin, epinephrine), substances released from activated platelets (platelet agonists like adenosine diphosphate—ADP, serotonin, thromboxane A_2_—TxA_2_), and vessel wall extracellular matrix constituents that come in contact with adherent platelets (collagen). Signal transduction is initiated at the binding receptors of platelet activators: GPVI for collagen, GPIba for vWF; protease-activated receptor-1 and -4 (PAR 1 and PAR 4) for thrombin, purinergic receptors 2Y_1_ and 2Y_12_ (P2Y_1_, P2Y_12_) for ADP, thromboxane receptor (TP) for TxA_2_; alpha 2A adrenergic receptor (alfa2A-AR) for epinephrine, and platelet activation factor receptor (PAF-R) for platelet activation factor (PAF) [106].

Agonists binding to platelet receptors initiates the activation process, starting with activation of phospholipase C (PLC) isoforms which hydrolyse phosphatidylinositol 4,5-bisphosphate (PIP_2_) producing second messengers—1,2-diacylglycerol (DAG) and 1,4,5-trisphosphate (IP_3_)—which trigger intracytosolic calcium elevation. IP_3_ binds to its receptor on dense reticular system (DTS) membrane and opens Ca^2+^ channels, producing an increase in cytosolic Ca^2+^ concentration [107]. High intracellular calcium is associated with platelet shape shifting, procoagulant surface exposure, secretion of platelet granular content, further activation of surface glycoproteins, activation of phospholipase A (PLA) and protein phosphorylation, all necessary for α_IIb_β_3_ integrin (aka GP IIb/IIIa) activation. DAG activates protein kinase C (PKC), a key player in platelet secretion and activation of GP IIb/IIIa. PLA releases arachidonate from membrane phospholipids, then TxA_2_ is synthetized, inducing further platelet recruitment and promoting thrombus formation [105].

Transient platelet activation is inhibited by both endothelial prostaglandin I_2_ or prostacyclin (PgI_2_) and nitric oxide (NO). PgI_2_ acts on its receptor (IP) and activates adenylyl cyclase (AC), increasing cyclic adenosine monophosphate (cAMP). NO acts on guanylate cyclase (GC), rising cyclic guanosine monophosphate (cGMP) levels. High cAMP and cGMP platelet levels are associated with the attenuation of cytosolic Ca^2+^ increase in response to agonists, an accelerated DTS uptake of intracellular Ca^2+^, impaired platelet secretion, and inhibition of α_IIb_β_3_ integrin activation. AC activity is inhibited by ADP via P2Y_12_ receptor and by epinephrine via alfa2A-AR, in order to facilitate platelet activation [105].

Upon activation, platelets granule content is released, further promoting adhesion and activation of other platelets. Activated platelets form a clump at the site of the vessel injury in a process called *aggregation*. Aggregation involves the formation of fibrinogen bridges in between platelets, following the activation of GPIIb-IIIa, a fibrinogen and vWf platelet transmembranary receptor [105].

## 4. Taurine and Platelet Function

Because taurine was seen as a non-patentable nutrient, the pharmaceutical industry has not shown much interest in its research. Most nutritionists have not paid much attention to it maybe because taurine represents a “semi-essential” or “conditional” amino acid, and not an essential one. Fortunately for the scientific world, as McCarty very well remarked, “physiologists have taken up the slack” in defining its role, followed by reports of a promising pharmacological action, especially in the recent years [108].

Platelets play a key role in the physiology and pathophysiology of hemostasis. The beneficial influence of taurine on the complex process of hemostasis may be primarily ascribed to its action on platelets. In this regard, one of the simplest tests that can be performed is assessing platelets taurine content, which depends on whole body taurine.

### 4.1. Taurine Content of the Platelets

Platelets are known to contain all essential amino acids. Taurine is present in platelets in a six times higher concentration than any other amino acid. From Maupin’s data, taurine platelet–plasma gradient is 440:1 [109]. Ahtee et al. suggested that taurine intra-platelet accumulation occurs against platelet–plasma concentration gradient. Platelet in vitro ability to accumulate radiolabeled taurine from the surrounding environment is based on the existence of an active transporter, which is responsible for generating and maintaining a high platelet–plasma concentration gradient [110]. A direct correlation, influenced by taurine intake, between platelet and plasma taurine concentrations has been reported in cats and humans [111,112,113]. A human study conducted in diabetic patients found a lower level of both plasma and platelet taurine in diabetic subjects compared to healthy controls, associated with a reduced uptake and an elevated taurine release from platelets. This has raised the question of whether there could be changes in the function of the two-carrier systems (with low and high affinity) that might lead to that reduction in platelet taurine content [114,115]. More recently, an animal study compared platelet taurine level in taurine-deficient dogs and taurine-sufficient dogs. A direct correlation between platelet and plasma taurine concentration has been established when data have been analyzed for each group. In the taurine-sufficient group, platelet taurine level was better correlated with its blood level than in the taurine-deficient group [116].

Platelet taurine concentration in animals or humans can be predicted by knowing plasma or whole-body taurine concentration. However, plasma taurine is not as a good predictor as platelet taurine level for platelet function, because platelets are able to accumulate taurine against a plasma–platelet concentration gradient [113,117,118]. The level of platelet taurine accumulation could be an important contributor to the inhibitory effects of the sulfur-amino acid on platelet function, as described further below.

### 4.2. Taurine Influence on Platelet Hemostatic Activity

Taurine and related compounds have been repeatedly pointed as modulating factors of platelet function [113,119], but the interest regarding their influence on blood coagulation dates from the early 1950s and 1960s [120,121]. Research on taurine effects over hemostasis started with the observation that bile acids (such as taurocholic, taurodeoxycholic, or taurochenodeoxycholic acid) and their salts elicit a considerable inhibition on platelet aggregation induced by various agonists (ADP, collagen, and others) [122,123,124,125]. It has been assumed that a probable impaired platelet aggregation in the upper segment of the gastrointestinal tract induced by biliary reflux might partly explain the poor hemostatic response in these otherwise normal human subjects [122].

These observations opened a new perspective on taurine and derivatives’ influence on the complex process of hemostasis, which was further greatly exploited.

For easier reading, a division based on animal and human studies has been established.

#### 4.2.1. Evidence from Animal Studies

Platelet function impairment plays a pivotal role in the onset and development of arterial thrombosis. Thus, finding new antithrombotic drugs, with or without minimal adverse effects is a subject of great interest. To date, animal studies provide substantiating evidence regarding the inhibitory action of taurine (and its related compounds) on platelet function, some of these studies even suggesting a putative antithrombotic potential of the sulfur-containing amino acid. The representative articles have been summarized and tabulated in Table 1.

One of the early experimental in vitro studies exploring the effect of taurine on platelet activity and reactivity was conducted by Kurachi et al. (1987) in guinea pigs. He demonstrated that taurine preincubation (40 nM) with platelet-rich plasma (PRP) from these animals can inhibit PAF-induced platelet aggregation. This sustained another important finding in the second ex vivo part of this study, showing a reduction in PAF or serotonin-induced bronchoconstriction following taurine administration (50 mg/kg, iv). Since PAF is involved in platelet degranulation with serotonin release, and since the bronchoconstrictor response to PAF has been noted to diminish the effect of a following prostacyclin or antiplatelet antibody pretreatment, it has been suggested that the observed suppressive action of the sulfur-amino acid on PAF-induced bronchospasm might be caused by an inhibitory effect on platelet aggregation [126].

El Tahir et al. (1987) demonstrated concomitantly that taurine may be involved in the regulation of prostaglandins (PGs) synthesis in various organs of female rats. Taurine administration (100 or 200 mg/kg/day) in drinking water for 6 weeks resulted in an elevation of aortic and uterine PgI_2_ and a diminution of uterine TxA_2_ release. Moreover, taurine incubation (0.4 and 0.8 mM) with tissues from 18-day pregnant rats enhanced uterine and aortic PgI_2_ release. These results globally suggested a potential benefit of taurine in those diseases presenting with a deficiency in PgI_2_ release [127].

A few years later, Hayes et al. (1989) showed in an ex vivo trial that taurine supplementation in cats’ diet (0.5 g taurine per kilogram) led to a significantly higher (140%) platelet aggregation threshold (PA_t_, defined as the amount in µg of collagen required to elicit 10% of the predetermined maximum aggregation in 1 mL PRP) than in taurine-depleted cats [113]. They also found that PA_t_ increase was associated with a higher concentration of platelet and plasma taurine, as well as platelet glutathione, the latter being known to diminish sensitivity to aggregating stimuli [128]. Arterial thromboembolism in cats with taurine-deficiency syndrome has been attributed to cardiomyopathy development, and it is apparently caused by mural thrombi formation and embolization from the left failing heart [21,129]. In their study, Hayes and colleagues speculated that taurine depletion in cats could actually induce both cardiomyopathy and thromboembolic phenomena [113]. On the other hand, Welles et al. (1993) reported reduced platelet aggregation and platelet serotonin release triggered by ADP, as well as an unchanged aggregation and mild increase of platelet serotonin release following collagen stimulation, in taurine-deficient versus taurine-repleted cats. The author noted that increased sensitivity to collagen and decreased responsiveness to ADP observed in taurine-deficient cats was quite surprising, considering that irreversible platelet aggregation following an initial sufficient stimulus is reached with dense granules ADP release [130].

At that time, another study showed that taurine (30 mg/kg/day, for 9 weeks) is effective in decreasing the elevated magnitude of ADP or thrombin-induced platelet aggregation in two-kidney-one-clip (2k-1c) Goldblatt renovascular hypertensive rats. However, taurine could not restore the increased level of aggregation in 2k-1c rats to that noted in normal rats; but co-treatment with enalapril (6 mg/kg/day, for 9 weeks) had a complementary effect, restoring aggregation magnitude in 2k-1c rats to that found in normal rats. Considering the quite similar results obtained by the authors on blood pressure and LVW/BW (left ventricular weight/body weight) ratio, it has been concluded that the two drugs, i.e., taurine and enalapril, may potentate each other and may be considered useful in hypertension treatment [131]. The same research group also demonstrated that taurine (100 mg/kg) can inhibit in vivo thrombosis generation and suppress ADP, collagen or thrombin-triggered aggregation (a reduction of platelet aggregation by a rate of 37.40%, 44.41%, and 37.87%, respectively, vs. controls) in rats. Moreover, concomitant treatment with taurine and neferine has been shown to be effective in reducing TxA_2_ generation in rat PRP, without affecting plasma PgI_2_ production, therefore suggesting a possible mechanism to underlie the effect of the two drugs administration on platelet aggregation and induced-thrombus formation [41].

The antiaggregant effect of taurine has also been noted in a rat model of hypercholesterolemia. Daily supplementation with 5% taurine in Sprague Dawley rats fed with a cholesterol diet for 4 weeks resulted in a suppression of whole blood platelet aggregometry, a method that has the advantage of being conducted under nearly physiological conditions. The maximum ADP (2 µM)-induced platelet aggregation at the point where aggregation dissociates was significantly decreased both in taurine vs. control group (14.36 ± 1.85 vs. 19.46 ± 3.20), and in the mixed-fed (cholesterol and taurine) vs. cholesterol-alone-fed group (14.20 ± 3.06 vs. 17.13 ± 3.72). The inhibition of platelet aggregation, along with the hypolipidemic and hypocholesterolemic effects of taurine revealed by this study indicate a possible favorable action of the sulfur-amino acid in the prevention of cardiovascular disease [132].

Using Born aggregometric method, we demonstrated that taurine (2% supplemented in drinking water) can decrease platelet aggregation in either normal rats, or in those concomitantly receiving a supraphysiological AAS (anabolic androgenic steroids) dose (10 mg/kg, weekly intragluteal injection, for 3 months) [133]. In a following thromboelastographic (TEG) experiment assessing shear elasticity of the clotting blood, we detected a slight tendency (without reaching statistical significance) of taurine to decrease maximal clot strength and stability in either normal or mixed treated group of rats [134].

Interestingly, there are data showing that the antiaggregant action of taurine can be translated to its derivatives. In 2002, Murina et al. resumed previous studies performed by her colleagues in humans and revealed novel antithrombotic properties of the chloramine derivative DT. In the first in vivo part of the experiment, she used an animal model of thrombosis (results already presented in the previous section and Table 1). The following ex vivo part of this study showed that intravenous injection of DT (6.8 mg/kg) led to a 50% decrease of ADP (10 µM)-triggered platelet aggregation in platelet-enriched plasma from the test group, compared to controls. The author concluded that the antithrombotic effects of chloramine derivatives of taurine may be attributed to their ability to down-regulate platelet activity [43]. To sustain the antiplatelet effect of DT, Murina et al. demonstrated, in an in vitro study using a kinetic nephelometric technique, that DT can also suppress the initial ADP-induced platelet aggregation (formation of small aggregates) in rabbit platelets, and that its effect may be ascribed to platelet sulfhydryl groups changes [135]. DT in a concentration of 10 µM reduced by half the intensity of small-angle light scattering aggregation triggered by 0.2 µM ADP in isolated rabbit platelets, while DT addition in a moderate concentration (10 millimoles/L) to rabbit blood was shown to markedly inhibit the impedance measured by whole blood aggregometry, triggered by 10 µM ADP [136]. A rank of efficacy in suppressing initial aggregation of isolated rabbit platelets was established for several taurine chloramine derivatives. DT, was found to be the most effective, providing a 50% decrease of PA at a concentration of about 0.2 mM, whereas N-chlorotaurine (CT) and N-chloro-N-methyltaurine (CMT) showed an inhibition of only 10% of PA induced by 10 µM ADP at a concentration of 0.5 mM. This behavior was explained through an enhanced reactivity of DT (compared with CT or CMT) for sulfhydryl groups on platelet surface. DT exhibited the greatest velocity constant (>10^3^ M^−1^ × s^−1^) when reacting with reduced glutathione comparing with NC (5.90 ± 1.12 M^−1^ × s^−1^) or CMT (3.20 ± 1.04 M^−1^ × s^−1^). Another important finding in this study was that the antiaggregant effects of taurine chloramines are potentiated by the presence of serum albumin (aggregation magnitude of taurine derivatives was 2-fold higher in the presence of albumin). Since albumin also exposes sulfur-containing groups, formation of protein-chloramine complexes could generate changes in protein conformation, providing a more effective interaction with platelets [137]. New derivatives of taurine chloramines have been later developed using a computational quantum-mechanic estimation (a computer calculative prediction) in order to enhance the charges of active chlorine, and therefore to increase the stability and reactivity of taurine derivatives for the thiol groups (such as those exposed by reduced glutathione, amino acids, peptides, or ADP receptors). N-acetyl-N-chlorotaurine and N-propionyl-N-chlorotaurine are examples of amido-derivatives of taurine chloramines with the greatest charge of active chlorine. They exhibited an elevated reactive ability (chemoselectivity) with respect to various sulfur-containing groups in amino acids and peptides and proved a firm inhibition of platelet aggregation triggered by ADP or collagen. Their effect on aggregation might be ascribed to the chemoselectivity and modification of the thiol group in P2Y_12_ ADP receptor, similar to that elicited by the main covalent inhibitors of platelets, the thienopyridine compounds (clopidogrel, prasugrel) [138]. Furthermore, an in vitro comparison between the antiaggregant effect of the amide analog of taurine chloramine, N-propionyl-N-chlorotaurine (PCT) and the alkyl analog, N-isopropyl-N-chlorotaurine (IPCT) was performed. PCT addition in a concentration of 1 mM on rabbit PRP suppressed platelet aggregation to a greater extent (38 ± 4.1%) than 1 mM of IPCT (60 ± 4.2%), following stimulation with 10 µM ADP. On the other hand, inhibition of platelet aggregation was similar when triggered with 32 μg/mL of collagen. The positive charge of active chlorine and the charge modulus of the nitrogen atom were 5- and 1.4-fold higher, respectively, in the amide than in the alkyl analog. The stronger platelet aggregation suppression of amide derivatives compared to alkyl analogs of taurine chloramines may in fact depend on the more efficient modification of the sulfhydryl group of ADP receptor [139].

#### 4.2.2. Evidence from Human Studies

There is a growing body of evidence depicting the influence of taurine on human platelet function, confirming the encouraging outcomes from animal studies.

Almazov et al. (1985) was one of the first to describe the effect of taurine on platelet aggregation, and to underline a possible mechanism of its action. Taurine incubation (25 nM) with human PRP resulted in a decrease of both spontaneous and induced (by 3.5 µM ADP) platelet aggregation rate, in the taurine treated platelets vs. control platelets (1.0 ± 0.3 vs. 2.1 ± 0.6, and 30.3 ± 5.8 vs. 62.8 ± 9.8, respectively). In the same in vitro study, taurine (25 mM) was also shown to enhance platelet Ca, Mg-ATPase activity by 45%, an effect proven to be mediated through the stimulatory action of taurine on calmodulin [140] (Table 2).

These findings are consistent with those reported by Raghu et al. (1982), who previously detected an in vitro dose-dependent inhibition of aggregation by taurine addition to platelets, when triggered by ADP, epinephrine, and collagen. This process seemed to involve a mechanism related to Ca^2+^ (calcium) ion translocation and was enhanced by increasing alkalinity [141].

Later, taurine influence on platelet aggregation has been assessed ex vivo. Hayes et al. (1989) demonstrated that taurine supplementation for 8 days in the diet of nonsmokers, normolipidemic, healthy male volunteers led to an increased plasma and platelet taurine concentration, as well as to a higher PA_t_ (an increase by 25%, or 72% following taurine administration in a dose of 400 mg/day, or 1600 mg/day, respectively) [113]. The percent increase of PA_t_ in this study was positively and relatively good correlated (r = 0.71, *p* < 0.005) with the percent increase of platelet taurine concentration, at both taurine doses. Furthermore, a decreased platelet TxB_2_ (thromboxane B_2_) release has been noted following stimulation of PRP from individuals supplemented with 1600 mg/day (for 8 days) with a constant amount of collagen (0.93 µg). Moreover, platelet GSH (glutathione) concentration analysis revealed an elevation of 34% in the taurine treated group (400 mg/day, for 8 days) versus controls, an important finding considering previously reported GSH inhibition on platelet aggregation [128,142]. All these results made the authors conclude that platelet taurine level depends on taurine intake, reflects the global taurine status of the organism, and certainly influences the magnitude of collagen-induced platelet aggregation. Taurine’s antiaggregant action may be partly ascribed to its sparing effect on platelet GSH pool [113].

Without showing an obvious effect *per se*, taurine has been found to markedly potentiate the in vitro inhibitory action of aspirin on collagen-induced platelet aggregation in PRP from healthy volunteers, in a dose-dependent manner [143]. This raised the question whether taurine might be useful in the future as a complementary therapy in patients treated with low-dose aspirin, aiming to decrease the thromboembolic risk [144]. Several years later, resuming their studies on the antiaggregant effect of taurine, Franconi et al. investigated its influence on platelets sampled from insulin-dependent diabetes mellitus (IDD) or non-insulin-dependent diabetes mellitus (NIDD) subjects [145,146]. They first noted a reduced level of both plasma and platelet taurine in IDD patients versus healthy subjects, and only a decrease of platelet taurine in NIDD patients. Preincubation of taurine (10 mM) with PRP from IDD patients shifted the whole dose-response curve of platelet aggregation to the right, depending on variable doses of arachidonic acid (from 0.2 to 1 mM), whereas the diagram remained unchanged in NIDD and in healthy patients. Additionally, an inverse dose-dependent relationship between taurine concentration and the magnitude of platelet aggregation has been revealed at a fixed agonist dose (0.6 mmol/L of arachidonic acid) in IDD patients. Following in vitro studies, in a second series of experiments conducted in IDD patients subjected to a taurine supplemented diet (intake of 1.5 g/day, for 3 months), an increase of both plasma and platelet taurine compared to base-line values was registered, as well as a reduced arachidonic acid-induced platelet aggregation. ED_50_ of arachidonic acid (representing the effective dose of the agonist necessary to reach a 50% magnitude of maximal aggregation) was significantly decreased (0.44 ± 0.07 mmol/L) in IDD patients compared to controls (0.77 ± 0.02 mmol/L) at the beginning of the experiment, while chronic taurine intake practically restored ED_50_ level (0.72 ± 0.04 mmol/L) to that of controls. Moreover, taurine supplementation shifted to the right the dose-response curve of platelet aggregation magnitude to increasing concentrations of arachidonic acid (up to 1 mmol/L) in IDD patients [145,146]. According to these data, the authors suggested that taurine’s potential to suppress platelet aggregation in diabetic patients may reduce complications, such as micro- and macroangiopathies, taurine being reported to exert a beneficial influence in retinopathy, renal damage, and cardiomyopathy [21,146,147,148].

On the other hand, no effect on platelet aggregation has been evidenced in a randomized, double blinded, crossover trial conducted in men with predisposition to type II diabetes mellitus. Taurine in a dose of 1.5 g daily supplemented in diet for two 8-week periods (and separated by 2 weeks of washout) exerted no influence on ADP-induced platelet aggregation. The lack of effect on aggregation was explained by an insufficient taurine plasma level reached following drug intervention [118].

Taurine influence on platelet function has been assessed in other clinical conditions, such as gestosis. When accompanied by edema, proteinuria, and hypertension (EPH gestosis), it has been linked to a hypercoagulative state [149,150], raising the risk of thrombosis [151,152]. Evidence suggests that platelet activation (measured by markers such as mean platelet volume, P-selectin, CD 63) is higher among woman with EPH gestosis. [152]. However, there are conflicting data regarding platelet aggregation in these patients, some studies reporting an increase, whereas other indicating a decrease of aggregation [153]. In their attempt to explain the coagulation abnormalities in EPH gestosis, Otani et al. (1992) evidenced an increased content of taurine in platelets and a higher in vitro taurine uptake into platelets, when the group of severe EPH gestosis patients was compared to normal pregnancy group, as well as with mild and moderate EPH gestosis group. The increase of taurine uptake by platelets was highly and positively correlated to the severity of EPH gestosis. Plasma taurine concentration was found unchanged in all groups of women [117]. In a following study, the same research group demonstrated that aggregation and platelet release response—measured as β-thromboglobulin (β-TG) and adenosine-triphosphate (ATP) discharge—were significantly reduced in EPH gestosis patients than in normal pregnant or non-pregnant women, when washed platelet suspension (WPS) obtained from these subjects was triggered with ADP (1 µM) or collagen (1 µg/mL). They also found that taurine inhibited platelet aggregation and reduced platelet ATP and β-TG release from WPS of non-pregnant women in a dose-dependent manner, following stimulation with ADP (0.5–1.5 µM), or collagen (0.5–1.25 µg/mL). Taurine’s antiaggregant effect paralleled with the increase of taurine concentration in platelets. A similar dose-dependent inhibition of platelet aggregation has been noted after triggering taurine-loaded WPS from non-pregnant women with 1 µM A23187 (a calcium ionophore). However, no effect of taurine in any doses on A23187-induced platelet aggregation has been registered following EDTA addition [154]. As A23187 is a non-physiological agonist able to increase platelet Ca^2+^ influx by changing membrane permeability [155], the lack of taurine’s effect on A23187-induced aggregation after EDTA addition made the authors assume that taurine might downregulate platelet Ca^2+^ influx [154]. This finding is in accordance with data reported by Atahanov et al. in the same year, with taurine (0.01 mol/l) being proven to blunt the in vitro platelet calcium response to PAF [156]. Overall, this study concluded that an increased capacity of platelets to uptake taurine in severe EPH gestosis may lead to a decrease in platelet aggregation, by reducing Ca^2+^ influx and diminishing platelets release response to agonists. However, caution is needed in drawing this conclusion. Decrease of aggregation in EPH gestosis may be also attributed to a refractory state (desensitization) of platelets, following their activation [152]. Therefore, the role of taurine in counterbalancing the hypercoagulative state associated to severe EPH gestosis remains to be reconsidered [154].

In another in vitro study, Miglis et al. showed that taurine at different concentrations (varying between 5 and 25 mM) suppressed to the same degree (10%) platelet aggregation induced by thrombin (1.0 U/mL), in PRP from healthy volunteers. No significant differences between taurine and control groups have been found when the extent of ADP (0.02 mM)-induced platelet shape change (ESC) was assessed. Moreover, only a slight tendency to decrease (but without reaching statistical significance) maximal clot strength and stability was registered when a thromboelastographic experiment (triggered by 0.6 U/mL thrombin and 6.25 mM CaCl_2_) was performed. Regarding the differences of the recorded results, the author speculated that ADP- induced aggregation is known to use other platelet receptors than ESC triggered by ADP. In addition, a 10% magnitude change of platelet aggregation generated by taurine may not be strong enough to induce a significant change in the overall clot strength measured by TEG. Moreover, the effect of Ca^2+^ added to initiate TEG testing may overcome the inhibitory effect of taurine on platelet activity [119].

The same beneficial effect of taurine on platelet aggregation was proven individually or in combination with caffeine in an in vitro study on PRP from healthy donors, suggesting that low concentrations of these compounds (lower than those usually found in energy drinks), may have a synergistic effect in reducing platelet activity [157].

**Table 1:** The characteristics of included animal studies.

Reference	Animal Subjects, Sex, Number of Animals per Group, Type of Experiment	Taurine or Related Compounds	Design of the Study (Taurine Dose, Time of Administration)	Platelet Aggregation Variation (Agonist)	Outcome from Other Assays of Platelet Function (Agonist), or from Animal Models of Thrombosis
Kurachi, M. et al., 1987 [126]	Guinea pigs, in vitro	Taurine	40 nM, 2 min before adding the agonist	↓ (PAF)	
Hayes, KC. et al., 1989 [114]	Cats, males and females with equal distribution, *n* = 6, ex vivo	Taurine	0.5 g T/kg diet (from the time of weaning to the age of 10–24 months)	↓ (↑PA_t_ by 140% in T-supplemented vs. T-deficient cats, when triggered with collagen)	↑ of platelet GSH concentration by 53% in T-supplemented vs. T-deficient cats
Ji, Y. et al., 1995 [131]	Rats (2k1c), *n* = 6, ex vivo	Taurine	30 mg/kg/day, for 9 weeks	- ↓ from 95.95 ± 2.13 (C group) to 87.63 ± 4.47 (T group) (ADP, 4 µmol/L) - ↓ from 88.22 ± 3.81 (C) to 74.92 ± 7.56 (T) (thrombin, 4 U/mL)	
Huang, HL. et al., 1995 [41]	Rats, *n* = 6, in vivo	Taurine	100 mg/kg		↓ of thrombosis wet weight by a rate of 47.82%, vs. controls
	Rats, *n* = 6, ex vivo	Taurine	100 mg/kg	- ↓ by a rate of 37.40% (T vs. C group) (ADP, 2 µmol/L) - ↓ by a rate of 44.41% (T vs. C group) (collagen, 0.05 mL/mL PRP) - ↓ by a rate of 37.87% (T vs. C group) (thrombin, 1 U/mL)	↓ platelet TxA_2_ release (ADP)
Park, IS. et al., 2007 [132]	Rats, *n* = 10, ex vivo	Taurine	5% in diet, for 4 weeks	- ↓ MAgr from 19.46 ± 3.20 to 14.36 ± 1.85 (T vs. C group) (ADP, 2 µM) - ↓ MAgr from 17.13 ± 3.72 to 14.20 ± 3.06 (Ch. + T group vs. Ch. alone fed group of rats) (ADP, 2 µM)	
Roşca, A. et al., 2013 [133]	Rats, males, *n* = 10, ex vivo	Taurine	2% in drinking water, for 3 months	↓ (ADP, 2.5 µM)	
Roşca, A. et al., 2013 [134]	Rats, males, *n* = 10, ex vivo	Taurine	2% in drinking water, for 3 months		N outcome for MA measured by TEG
Murina, M.A. et al., 2002 [135]	Mice, in vivo	DT	- 3.4–6.8 mg/kg - 6.8 mg/kg		- ↓ mortality rate from 96% in controls to up to 10% in DT group (ADP, 300 mg/kg) - ↑ survival rate from 16% in controls to 64% in DT group (administered mixture: 15 mg/kg collagen and 8.6 mg/kg epinephrine)
	Mice, male, ex vivo	DT	6.8 mg/kg, i.v	↓ by a rate of 50% (DT vs. C group) (ADP, 10 µM)	
Murina, M.A. et al., 2007 [136]	Rabbits, in vitro	DT	10 µM	↓ I_SALS_ by half (ADP, 0.2 µM)	
	Rabbits, in vitro	DT	10 millimoles/L	↓ markedly the impedance measured by whole blood aggregometry (ADP, 10 µM)	
Kaptanoglu, L. et al., 2008 [44]	Rat, *n* = 10, in vivo	TL	10 mg, or 20 mg, i.v; Heparin (100 antiXa ICU/2 mL/kg, nadroparin calcium)		↓ of thrombus weight by a rate of 42 % vs. C (but significantly higher than that in heparin treated group).
Murina, M.A. et al., 2009 [137]	Rabbits, in vitro	- DT - CT, or CMT	- 0.2 mM - 0.5 mM	- ↓ I_SALS_ by 50% (ADP, 10 µM) - ↓ I_SALS_ by 10% (ADP, 10 µM)	
Murina, M.A. et al., 2014 [139]	Rabbits, in vitro	- PCT - IPCT	- 1 mM - 1 mM	- ↓ to 38 ± 4.1% vs. baseline (100%) (ADP, 10 µM) - ↓ to 60 ± 4.2 % vs. baseline (100%) (ADP, 10 µM)	

Abbreviations: PAF—platelet aggregating factor; *n*—number of subjects per group; T—taurine; PA_t_—the amount in µg of agonist required to elicit 10% of a predetermined maximal aggregation in 1 mL PRP; PRP—platelet rich plasma; GSH—glutathione; 2k1c—two-kidney-one-clip Goldblatt renovascular hypertensive rats; C—controls; TxA_2_—thromboxane A_2_; ADP—adenosine diphosphate; MAgr—the maximum platelet aggregation at the point where aggregation dissociates; Ch.—cholesterol; MA—maximal clot strength and stability; TEG—Thromboelastography; DT—N,N-dichlorotaurine; I_SALS_—intensity of small-angle light scattering aggregation; CT—N-chlorotaurine; CMT—N-chloro-N-methyltaurine; TL—Taurolidine; PCT—N-propionyl-N-chlorotaurine; IPCT—N-isopropyl-N-chlorotaurine; ↑—up regulation; ↓—down-regulation; N—neutral effect.

**Table 2:** The characteristics of included human studies.

Reference	Human Subjects, Sex, Number of Individuals per Group, Type of Experiment	Taurine or Related Compounds	Design of the Study (Taurine Dose, Time of Administration)	Platelet Aggregation Variation (Agonist)	Outcome from Other Assays of Platelet Function (Agonist)
Almazov, V.A. et al., 1985 [140]	Human platelets, *n* = 10, in vitro	Taurine	25 nM	↓ by half (ADP—3.5 µM)	
	Human platelets, *n* = 5, in vitro	Taurine	25 nM		↑ of platelet Ca, Mg-ATPase activity by 45%
Hayes, K.C. et al., 1989 [113]	Healthy volunteers, male, *n* = 5, ex vivo	Taurine	400 mg/day, for 8 days	↓ (↑PA_t_ by 25% in T group vs. controls, when triggered with collagen)	↑ of platelet GSH concentration by 34%
	Healthy volunteers, male, *n* = 5, ex vivo	Taurine	1600 mg/day, for 8 days	↓ (↑PA_t_ by 72% in T group vs. controls, when triggered with collagen)	↓ platelet TxB_2_ release (collagen, 0.93 µg)
Franconi, F. et al., 1994 [145] and Franconi F. et al., 1995 [146]	- Human platelets from IDD patients, six experiments, in vitro - Human platelets from IDD patients, four experiments, in vitro	Taurine	- 10 mM - increasing dose (10^−6^, 10^−5^, or 10^−2^ mol/L)	- ↓ (arach. ac, various concentrations—from 0.2 to 1 mM) - ↓ (arach. ac, 0.6 mmol/L)	
	- IDD patients, *n* = 17, ex vivo - IDD patients, *n* = 35, ex vivo	Taurine	1.5 g/day, for 3 months	- ↓ (↑ED_50_, from 0.44 ± 0.07 mmol/L to 0.72 ± 0.04 mmol/L arach. ac) - ↓ (arach. ac, various concentration—up to 1 mmol/L)	
Spohr, C. et al., 2005 [118]	Men with predisposition to type II diabetes mellitus, *n* = 9, ex vivo	Taurine	1.5 g/day, for two 8-week periods (separated by 2 weeks of washout)	N outcome (TC: 3.86 ± 3.25 µmol/l for T group; 3.86 ± 2.21 µmol/l for placebo group)	
Namba, K. et al., 1992 [154]	Human platelets from non-pregnant women, *n* = 5, 10 experiments, in vitro	Taurine	increasing dose (6.25, 25, or 50 mM)	↓ with 25.6% to 42.4% (ADP, 0.5–1.5 µM), and with 29.5% to 36.7% (collagen, 0.5–1.25 µg/mL)	- ↓ ATP release response with 29.2% to 61.1%–triggered by ADP (0.5–1.5 µM), and with 54.5% to 57.9%—triggered by collagen (0.5–1.25 µg/mL) - ↓ β-TG release response with 21.8% to 48.1%—triggered by ADP (0.5–1.5 µM), and with 29.9% to 41.2%—triggered by collagen (0.5–1.25 µg/mL)
	Human platelets from non-pregnant women, *n* = 5, 5 experiments, in vitro	Taurine	increasing dose (6.25, 25, or 50 mM)	- ↓ with 46% to 69.4% (A23187, 1 µM) - N outcome (A23187, 1 µM, following 1 mM EDTA addition)	
Miglis, M. et al., 2002 [119]	Human platelets, 5 different donors, in vitro	Taurine	increasing dose (5 to 25 mM)	↓ by 10%, for each T dose (thrombin, 1.0 U/mL)	
	Human platelets, 5 different donors, in vitro	Taurine	5 or 25 mM		N outcome for ESC (0.02 mM ADP)
	Human platelets, 5 different donors, in vitro	Taurine	25 mM		N outcome for MA measured by TEG
Murina, M.A. et al., 2007 [136]	Platelets from healthy donors, in vitro	Taurine	10 mM	N outcome (ADP, 10 µM)	
	Platelets from healthy donors, in vitro	Taurine and NaOCl	10 mM and 1 mM, respectively	↓ (↑MI by 1.7 times in the mixed treated vs. NaOCl alone group) (ADP, 10 µM)	
	Platelets from healthy donors, in vitro	DT	0.25 mM	↓ (↑MI to 40 ± 7) (ADP, 10 µM)	

Abbreviations: *n*—number of subjects per group; ADP—adenosine diphosphate; Ca, Mg-ATPase—calcium, magnesium ATPase; PA_t_—the amount in µg of agonist required to elicit 10% of a predetermined maximal aggregation in 1 mL PRP; GSH—glutathione; TxB_2_—thromboxane B_2_; IDD—insulin-dependent diabetes mellitus; arach. ac—arachidonic acid; ED_50_—effective dose _50_, or the amount of agonist necessary to reach the 50% magnitude of maximal aggregation; PRP—platelet-rich plasma; PPP—platelet-poor plasma; TC—the threshold concentration, or the lowest concentration in µmol/l of ADP required to elicit irreversible aggregation (with a difference of at least 80% in light transmission between PRP and PPP); ATP—adenosine triphosphate; β-TG—β-thromboglobulin; A23187—calcium ionophore; EDTA—ethylenediaminetetraacetic acid; T—taurine; ESC—extent of platelet shape change; MA—maximal clot strength and stability; TEG—thromboelastography; NaOCl—sodium hypochlorite; vs.—versus; MI—magnitude of aggregation inhibition, standardized to control; ↑—up regulation; ↓—down-regulation; N—neutral effect.

In 1997 and 1998, Roshchupkin and collaborators published several studies regarding the antiplatelet action of chloramine derivatives, such as DT [158,159,160,161]. DT was shown to inhibit platelet-dense granule secretion, suppress spontaneous and induced aggregation, induce disaggregation of aggregated platelets, and exert systemic antithrombotic effects [158,159,160,161]. Additionally, DT elicited similar efficacy as acetylsalicylic acid (ASA) and ticlopidine in inhibiting secondary platelet aggregation, and even more potency than those drugs in suppressing primary platelet aggregation [158]. More recently, Murina et al. showed that taurine alone in a concentration of 10 mM failed to change platelet aggregation in PRP from healthy donors, while in combination with 1 mM sodium hypochlorite (NaOCl) it potentiated the antiaggregant effect of NaOCl by 1.7 times, following stimulation with 10 µM ADP. Further, incubation of PRP with DT (0.25 mM) inhibited platelet aggregation to the same extent as 1 mM NaOCl [136]. Another finding of this study, obtained from experiments not performed on humans but on animals (rabbit PRP), showed that biogenic chloramines are able to elicit a high initial selectivity for platelet surface binding (result expressed as a higher rate constant of chloramines interaction with platelet receptors, than that achieved when attaching to plasma proteins receptors) [136]. The pronounced antiaggregant effect of DT described in these studies performed by Murina and collaborators [135,136,158,159,160,161], the apparent safety of its therapeutic doses [136], the high stability during a long-term period of storage (4 months), and the notable selectivity in reaction with platelets, led the authors suggest that there are good grounds to believe that DT could be an adjuvant drug in future antiplatelet therapy [136].

Overall, although human studies describing the effect of taurine and its derivatives on platelet function bring less information than animal experiments, they are consistent with them and provide additional insights into their mechanisms of action, thus contributing to a broader picture of their beneficial effects.

Still far from being elucidated, the potential mechanisms underlying the overall inhibitory influence of taurine and its derivatives on platelet activity can be summarized as follows: decreased platelet TxA_2_ [41] and TxB_2_ production [113]; suppression of platelet cyclooxygenase activity [144,158]; stimulation of calmodulin-mediated platelet Ca, Mg-ATPase activity [140,162], attenuation of platelet Ca^2+^ influx [141,154] and suppression of intraplatelet Ca^2+^ response to activating agonists [156]; platelet stabilization against PAF [126,143,156]; suppression of β-TG and ATP release response to agonists, as markers of discharge from alpha and dense platelet granules [154]; preservation of platelet glutathione pool [113]; increased affinity of covalent inhibitors (e.g., DT, PCT, IPCT) to molecular targets, i.e., sulfur-containing groups on platelet surface (such might be the thiol group of P2Y_12_ ADP receptor) [135,136,137,138,139]; and pronounced enhancement of hydrogen sulfide (H_2_S) plasma level [163,164,165,166], H_2_S being known to inhibit platelet activation and aggregation [167,168,169,170,171,172]. Taurine may also interfere with platelet activity by generating complementary processes such as an increase of the endothelial NO release [173,174,175,176], decrease of epinephrine and norepinephrine circulant level [177,178,179], suppression of CD147-dependent MMP-9 pathway on ischemic brain endothelium [54], reduction of serum TxB_2_ [175], decrease of TxA_2_ and TxB_2_ release from various organs [127,180,181,182,183], while increasing PgI_2_ production [127,183]. Through all these mentioned pathways taurine and its derivatives are thought to render platelets more stable against a large variety of aggregating agonists, both physiological (ADP, thrombin, collagen, epinephrine, and others) [113,119,132,146] or non-physiological (e.g., A23187) [154].

## 5. Concluding Remarks

Based on a critical analysis of all existing data, we can conclude that there are good reasons to believe that taurine and its derivatives should receive more attention from the scientific world for their inhibitory action on platelet activity and their possible antithrombotic potential. In vivo, ex vivo and in vitro animal or human studies have provided complementary information and succeeded into shaping taurine and its analogues’ antiplatelet profile to a certain extent. However, caution is needed when promoting bold conclusions, as there are some important study limitations, mainly related to their experimental design, statistical power, or the relative scarcity of the presented mechanisms. The heterogenicity of the assays, treatment protocols, or study populations makes data analysis and pooling difficult. More comprehensive animal outcomes are certainly needed, to validate the existing findings and to give new perspectives on intimate mechanisms, before extrapolated to humans. However, there is one limitation that seems to be very difficult to cross, and that is carrying out prospective human studies on thrombosis outcome. Taurine-mediated protection against pathologies associating thrombotic diathesis, such as diabetes, traumatic brain injury, or acute ischemic stroke represents an argument that encourages further research of this fascinating nutrient, that seemingly displays permissible side effects. Nowadays, taurine is approved in Japan as a therapeutic agent for heart failure treatment, and only the lack of large-scale phase 3 clinical trials restricts taurine use as a therapeutic agent in several other pathologies for the treatment of which it has been shown to be effective (hypertension, atherosclerosis, stroke, neurodegenerative diseases, metabolic diseases, e.g., diabetes mellitus, and others). The discovery of taurine derivatives with higher stability and selectivity for sulfhydryl groups on platelet surface (e.g., DT or amido-derivatives), is another valuable step, which may be followed in the next years by the synthesis of new taurine chloramines with more specificity for the reactive sulfur-containing chemical groups on platelets, such as those found on ADP, collagen, or glycoprotein IIb/IIIa receptors. Searching for new, alternative antithrombotic drugs with minimal side effects (i.e., low risk of bleeding), and with a long-term inhibition on platelet activity (conferring the possibility of low dose administration), is probably currently the main goal in this area of interest. The question remains whether taurine, this semi-essential, commonly found, and highly beneficial amino acid (or its derivatives) will be able to face this challenge in the future. We shall see.
